# Evolution of Advanced Practice Provider Fellowship Training in Neurology Over 10 Years

**DOI:** 10.1212/NE9.0000000000200173

**Published:** 2024-10-31

**Authors:** Joel C. Morgenlander, Lesli Ann Simers, Kelly White, Bryan D. Walker

**Affiliations:** From the Department of Neurology (J.C.M., L.A.S., K.W.), Duke University, Durham, NC; and EMD Serono (B.D.W.), Inc., Boston, MA.

## Abstract

We are pleased to present our 10-year experience with our 1-year neurology advanced practice provider (APP) fellowship. The Duke fellowship was started by 1 author (J.C.M.) with institutional and departmental support as a response to 2 issues in our Neurology Department. The rationale for developing neurology APP fellowships nationally and locally are reviewed.

## National Factors Necessitating Advanced Practice Provider Training

The growth of nurse practitioner (NP) and physician assistant (PA) practice started in the 1960s to supplement issues with physician workforce.^1^ In addition, coverage issues such as work hour restrictions for residents could be aided by advanced practice providers (APPs) in practice. NP fellowships were encouraged by an Institute of Medicine report suggesting the need to improve clinical skills in areas of need and support better retention for these providers.^2^ Development of additional training opportunities were constructed first for primary care. A national survey of PA fellowship program directors showed programs were developed to offset issues with resident numbers and work hours, expanding clinical services and research capabilities.^3^ Over 90% of the PAs trained through the programs represented in this survey were employed within 2 months of their training completion speaking to the value of such training. Studies have shown improved job satisfaction among NPs with postgraduate training.^4^ More specifically in Neurology, survey and focused group evaluation of neurology APPs showed a need for improved neuroanatomy knowledge, advanced neurology examination skills, and differential generation. Inpatient APPs also needed more EMR skills, procedural exposure, and ability to care for complicated patients.^5^

## Local Factors Prompting an APP Neurology Fellowship

Our neurology practice at Duke faced several issues. Access to new patient appointments varied in our divisions from a minimum of 2.5 months to a maximum of 12 months. Our primary care partners and outside referral resources were struggling with the delay, and we were very concerned about the adverse outcomes from patient delay in diagnosis and treatment. Most APPs came to us with little or no neurology experience. Because APP training in either PA or NP training programs have little if any specific neurologic education and clinical exposure,^4,6,7^ APPs got on the job training mainly by observing their supervising neurologists. They were not afforded the opportunity to learn neurology in a more comprehensive manner and thus often were not confident in the neurologic model of evaluation including localization, formulation, differential diagnosis, and plan. This led to frustration among APPs, lack of trust by their supervising neurologists, and ultimately poor retention. There was no standardization to the process of onboarding between divisions. The “lesion” we believed was insufficient neurologic knowledge base and diagnosis and treatment skills on the part of the APPs. This was no fault of the APPs, just an understandable result of the construct of their original training. In addition, many of neurology APPs had little or no exposure to different areas of clinical neurology and so did not know whether a certain area of neurology would be best suited for their employment. They would be hired directly into 1 specialty, often right after graduation.

## APP Neurology Fellowship

Duke Neurology created a 1-year APP Fellowship in Neurology.^8^ The fellowship was created based on the substantial educational experience of 1 author (J.C.M.) including service as Neurology residency director for 18 years. There was no needs survey performed other than the clear wish to develop more comprehensive education and exposure to clinical neurology. We patterned the year loosely after a junior year of neurology residency with month long blocks taking the APP fellow into general neurology, specialty outpatient neurology and inpatient services (general neurology, stroke, and consultation neurology). The APPs are treated as learners and present every patient to either a supervising APP or neurologist. Each rotation has a rotation coordinator who is either an APP or neurologist. The coordinator arranges the schedule, helps to evaluate the trainee, and works with the program leaders to create goals and objectives for each rotation. We use the Accreditation Council for Graduate Medical Education (ACGME) core competencies as our outline for rotations.

Didactics are piggy-backed onto existing Department lecture series including Grand Rounds, the resident noon lecture series, and a weekly Departmental Interesting Case Conference. In addition, the APP fellows meet weekly with either the program director (J.C.M.) or the associate program director (L.S.) to review a list of core topics including neuroanatomy review, common neurologic clinical presentations, neuroimaging, professionalism issues, and individual cases the trainees have seen. We leverage the excellent basic neuroscience education program available on Coursera to aid in basic neuroscience education.^9^ The American Academy of Neurology journal, Continuum, is used as learning material for each rotation in addition to a standard neurologic textbook. Eventually, the Duke APP Fellowship started hosting quarterly journal clubs where each trainee would present article reviews being coached by a past APP fellow who is herself a doctor of epidemiology. Finally, APP fellows attend national and regional conferences and present a poster at 1 meeting. Clinical tests in the form of localization exercises and vignettes are given to the trainees to ensure progression in knowledge base. These quizzes developed over time to include both outpatient and inpatient scenarios.

## Recruitment

We advertise the program on the Duke Neurology website.^10^ At times we have advertised at other locations including local PA and NP schools and have given presentations at their respective informational fellow days reviewing post graduate training options. We learned that PA and NP programs graduate at different times of the year and eventually staggered our start time for our 2 fellows so they would not hit rotations at the same time. Applications include a curriculum vitae, 3 letters of recommendation, and a statement as to why the applicant wishes to train in neurology. Those that might be considered for interview are called by the program director (J.C.M.). If believed to be a potential candidate, applicants are invited to visit Duke for a tour and to meet with program leadership and current fellows. Hiring decision is made by the group including at a minimum 1 neurologist and 3 APPs. We base the salary level at the institutional postgraduate year 1 salary. Our APP fellows are treated as learners and thus paid less than the practicing APPs. Salary has not been an issue with our trainees. The hope is if training goes well and positions are available, the APP fellow will continue on at Duke.

## Challenges With Starting APP Training Programs

As with most new endeavors the first challenge is money. We had to convince our health system leadership that the case for the program was worthwhile. It was helpful that access problems and retention of our APPs were on the radar screen. It also helped that we decided because the fellows were going to treated as learners (i.e., generally not billing or seeing patients independently), we would pay them at an intern salary rate which was approximately two-third of the new hire APP rate. If pushed other options exist such as decreased training time. However, we believed the more complete training gained in a year program would pay dividends in APP confidence and practice skills. Because there is no training license available, APPs are credentialed as providers. One could schedule additional patients on the templates of those who train the APPs and have the APPs see those patients (staffing them with the supervising clinician), thus providing additional revenue and access.

We have tried to be careful to have our Neurology APP fellows blend into clinical rotations to avoid any conflicts with clinical volume seen by the Neurology residents. Because the APP fellows may be seeing the patients scheduled for our staff APPs, there is always an ability to be flexible in clinic scheduling. We very rarely have had any conflict when 2 trainees might show up in the same clinic with limited faculty, and it has not been an issue. We know this from ACGME survey results. On inpatient services, the Neurology APP fellows follow their own group of patients and write notes as appropriate being supervised by a staff APP. This is actually a help to the residents because the APP fellows are part of the coverage mix. Because our practice is multidisciplinary, we believe it is to the advantage of our Neurology residents to learn about APP training and practice as part of their education. When the APP fellowship started we believed it was important to present the fellowship to the residency program not only to explain the training but to educate the neurology residents about APP primary training and practice because APPs would be part of their education and practice. Medical student rotations were not affected by this program because the students follow inpatients with resident supervisors and see outpatients independently.

We also discussed the need for the APP fellowship at a faculty meeting to be sure questions were answered. Faculty buy-in is crucial as they are part of the APP education team. It was important to find faculty and/or APPs to be rotation coordinators because they are on the front line in clinic for any scheduling changes and would be in the best place to help the APP fellow with clinic scheduling and trouble-shooting time away. This could also be done by 1 dedicated leader from the APP fellowship.

## Outcomes

We have graduated the 19 APP fellows who have completed their training (1 fellow left for military service). The goal was to hire all who trained at Duke and this was discussed with the trainees during recruitment although guarantees could not be given. Fifteen of the 19 APP fellows were hired by our department (79%). Two fellows moved to another city because of family issues, 1 was offered an outpatient position but preferred to secure an inpatient position outside of our practice and 1 APP found a community position in the state. For comparison, in that same time period we graduated 47 residents of which 8 were hired (17%). Many of the first trained APP fellows still work at Duke Neurology. Duke APP fellows have worked on both inpatient and outpatient services and at this point in every departmental division. We have also sent our APP fellows to work in our sister hospitals to assist in staffing. When hired, we still ramp up templates over the first few weeks to give the new APP a chance to acclimate to practice but believe they have felt more comfortable, more quickly than those without the exposure.

Each time we hired an APP for our outpatient practice our access would improve through 2 mechanisms. We were able to change faculty templates to increase the numbers of new patients seen since the APP was seeing many of the returns. In addition, for several of our divisional practices, our APPs would see new patients as well. This was especially true for headache, sleep, and memory disorders. Our department APPs saw 201 new patients and 5,480 return patients in fiscal year 2015. In fiscal year 2023, our department APPs saw 3,351 new patients, 15,890 return patients, and 2,218 procedural patients (mainly Botox for headache, Botox for spasticity, and lumbar punctures).

## Survey of Graduated APPs

We surveyed the first 10 graduated APPs about the program. All APPs were interested in neurology before PA/NP school. Six APPs had prior neuroscience experience (2 BS degree, 1 MS degree, 1 PhD, and 6 with work experience). Seven of the 10 had neurology didactics in PA/NP school favoring those in PA programs. Six of the 10 APPs had a rotation in neurology in PA/NP school, most of 4 weeks duration. Nine of the 10 APPs felt the fellowship training length was just right with 1 feeling it was too short. Our typical split of time between supervising neurologist: supervising APP is 2:1 and all thought that split was good. All APPs felt they had adequate help finding a position post training.

## Survey of Supervising Neurologists

We surveyed the 12 supervising neurologists who worked with our APP graduates. Nine had worked with APPs before. Eleven had a positive experience with the APPs while 1 was neutral about the experience. Eleven neurologists felt they had an improved sense of trust with the graduates compared with other APPs and 11 felt the APP graduates had better history and neurology examination skills and were better able to order appropriate testing and consults than other APPs. Nine neurologists felt the graduate had improved their practice overall (3 were neutral).

## Conclusion

Neurology training fellowships help improve access to patients, create a pathway for APP onboarding, improve retention and support supervising neurologists through the improved skills of the APPs. The investment in training is already being made by current onboarding processes with slow ramp up of neurologically untrained APPs and studies have shown higher job satisfaction with additional APP training. Because of all of the issues discussed, it has been recommended that postgraduate training programs are the onboarding method of choice.^11^ Many other Neurology programs have started APP training programs and the program directors are currently meeting nationally. Work has begun on developing entrustable professional activities on which to base curriculum development. This hopefully would lead to a certification test for the graduates. The Neurology APP Fellowship at Duke has met our original goals for the program to improve access to patients and APP retention. We encourage others to consider the benefits of APP fellowship training programs in neurology.

## Appendix Authors

**Table:**

Name	Location	Contribution
Joel C. Morgenlander, MD	Department of Neurology, Duke University, Durham, NC	Drafting/revision of the manuscript for content, including medical writing for content; major role in the acquisition of data; study concept or design; analysis or interpretation of data
Lesli Ann Simers, NP	Department of Neurology, Duke University, Durham, NC	Drafting/revision of the manuscript for content, including medical writing for content; study concept or design
Kelly White, PA	Department of Neurology, Duke University, Durham, NC	Drafting/revision of the manuscript for content, including medical writing for content; study concept or design
Bryan D. Walker, MHS, PA-C	EMD Serono, Inc., Boston, MA	Drafting/revision of the manuscript for content, including medical writing for content; major role in the acquisition of data; study concept or design; analysis or interpretation of data
